# Exploring the Innate Immunological Response of an Alternative Nonhuman Primate Model of Infectious Disease; the Common Marmoset

**DOI:** 10.1155/2014/913632

**Published:** 2014-07-22

**Authors:** M. Nelson, M. Loveday

**Affiliations:** Biomedical Science Department, DSTL, Porton Down, Salisbury SP4 0JQ, UK

## Abstract

The common marmoset (*Callithrix jacchus*) is increasingly being utilised as a nonhuman primate model for human disease, ranging from autoimmune to infectious disease. In order to fully exploit these models, meaningful comparison to the human host response is necessary. Commercially available reagents, primarily targeted to human cells, were utilised to assess the phenotype and activation status of key immune cell types and cytokines in naive and infected animals. Single cell suspensions of blood, spleen, and lung were examined. Generally, the phenotype of cells was comparable between humans and marmosets, with approximately 63% of all lymphocytes in the blood of marmosets being T cells, 25% B-cells, and 12% NK cells. The percentage of neutrophils in marmoset blood were more similar to human values than mouse values. Comparison of the activation status of cells following experimental systemic or inhalational infection exhibited different trends in different tissues, most obvious in cell types active in the innate immune response. This work significantly enhances the ability to understand the immune response in these animals and fortifies their use as models of infectious disease.

## 1. Introduction

The common marmoset (*Callithrix jacchus*), a New World monkey (NWM) species is a small, arboreal nonhuman primate (NHP), native to the Atlantic Coastal Forest in Northeast Brazil and parts of South East Brazil. In recent years the common marmoset has become more widely used in applied biomedical research, and an increasing body of evidence suggests the physiological and immunological responses to biological insults are similar between marmosets and humans [1]. In the field of infectious disease, the marmoset is primarily being investigated as an alternative NHP model to complement the more traditionally used Old World monkeys (OWM) (e.g., rhesus and cynomolgus macaques). Evolutionarily, both NWM and OWM sit within the simiiformes infraorder of the suborder* Haplorhini* of primates [2]. Marmosets sit within the family Callitrichidae of the Platyrrhini parvorder, while OWM sit within the Cercopithecidae family of the Catarrhini Parvorder. Marmosets therefore are separated from Old World monkeys by one ancestral step and are a lower order primate.

Marmosets have been used to model the infection syndrome caused by a number of public health pathogens including Lassa virus [3], Hepatitis C virus [4], Dengue virus [5], Herpesvirus [6], Junin virus [7] Rift Valley Fever [8], and SARS [9]. Marmosets have also been used to model a number of biodefense pathogens including Eastern Equine Encephalitis virus [10],* Bacillus anthracis* [11],* Francisella tularensis* [12, 13],* Burkholderia pseudomallei* [14], Marburg haemorrhagic fever virus [15, 16], Ebola haemorrhagic fever virus [16], and Variola virus [17]. The utility of marmosets to assess medical countermeasures has also been demonstrated; a vaccine has been tested for Lassa fever [18] and the efficacy of ciprofloxacin and levofloxacin has been tested as postexposure therapies for anthrax and tularemia, respectively [19, 20].

In order to exploit these models fully and to allow meaningful comparison with the human condition, the response of the immune system to infection/therapy needs to be characterised and understood. Generally, NHPs have a close molecular, immunological, reproductive, and neurological similarity with humans making them ideal surrogates for humans and the study of infectious diseases. There is a high level of gene homology between humans and NHPs which underlies physiological and biochemical similarities. Similarities at the genetic level extend to the phenotypical level making NHPs well suited to modelling pathophysiological responses in man [21]. Immunologically, there is a high degree of homology between humans and marmosets [22]. The similarity of various immunological factors produced by humans and marmosets has been investigated at both the genetic and protein levels. There is at least 95% homology between human costimulatory molecules (e.g., CD80, CD86 etc.) and those of marmosets [23]. Also the immunoglobulin and T-cell receptor repertoire of humans and marmosets show at least 80% homology [24, 25].

Currently, the availability of commercial reagents specifically designed for the marmoset is limited although a number of antibodies designed for use with human samples have been shown to cross-react with leucocytes from marmoset blood [26–28]. However, these reagents have not been exploited to investigate the immune response to infectious disease. To date, investigation of the immune response in marmosets has primarily been achieved using pathogen-specific antibodies to determine the serological response using ELISA such as in the smallpox, Dengue, Rift Valley Fever, and Herpes models [5, 6, 8, 17] or by immunohistochemistry to identify, for example, CD8+, CD3+, CD20+ cells, and IL-6 in the smallpox model [17]; neutrophils and macrophages in the Herpes model [6]; or CD3+ and CD20+ cells in the Lassa model [3].

The work presented here focuses on understanding the immune profile of the naive marmoset as well as identifying and quantifying the immune response to infectious disease. The aim of this work is to determine key changes and identify correlates of infection or protection.

## 2. Materials and Methods

### 2.1. Marmosets

Healthy sexually mature common marmosets (*C. jacchus*) were obtained from the Dstl Porton Down breeding colony and housed in vasectomized male and female pairs. The Dstl colony was established during the 1970s and is a closed colony with a stable genotype. Animals included in these studies were mixed sex pairs, between 18 months and 5 years old and weighing between 320 g to 500 g. All animals were allowed free access to food and water as well as environmental enrichment. All animal studies were carried out in accordance with the UK Animals (Scientific Procedures) Act of 1986 and the Codes of Practice for the Housing and Care of Animals used in Scientific Procedures 1989. Animals were challenged with an intracellular pathogen by either the subcutaneous or inhalational route and were humanely killed at various time points after challenge. Prior to the infection study, animals were bled to determine baseline immunological parameters. Studies were performed to establish infection models in order to evaluate the efficacy of suitable therapies for transition ultimately to the clinic.

### 2.2. Flow Cytometry on Leucocyte Populations

Blood and tissue samples were homogenised to provide single cell suspensions [12]. Red blood cells were lysed, and the mixed leucocyte population was washed and stained with various combinations of the following fluorescent antibody stains: CD3 (SP34-2), CD8 (LT8), CD11c (SHCL3), CD14 (M5E2), CD16 (3G8), CD20 (Bly1), CD45RA (5H9), CD54 (HCD54), CD56 (B159), CD69 (FN50), CD163 (GHI/61), and MCHII (L243) (BD Bioscience, Insight Bioscience, AbD serotec). Samples were fixed in 4% paraformaldehyde for 48 hrs at 4°C and analysed by flow cytometry (FACScanto II BD) within 72 hours of staining.

Levels of circulating cytokines and chemokines were also quantified in the blood of marmosets from the Dstl colony using human multiplex kits available commercially (BD cytokine flex beads and the Luminex system). These systems show significant cross-reactivity with the marmoset suggesting a high degree of conservation between the two species for IL-6, MIP-1*α*, MIP-1*β*, and MCP-1 [29]. However, for other cytokines that are pivotal in the innate response, TNF*α* and IFN*γ* reagents were obtained from U-CyTech Biosciences and Mabtech AB, respectively, due to a lack of cross-reactivity observed within the kit obtained from BD [13].

## 3. Results and Discussion

**Figure 1:**
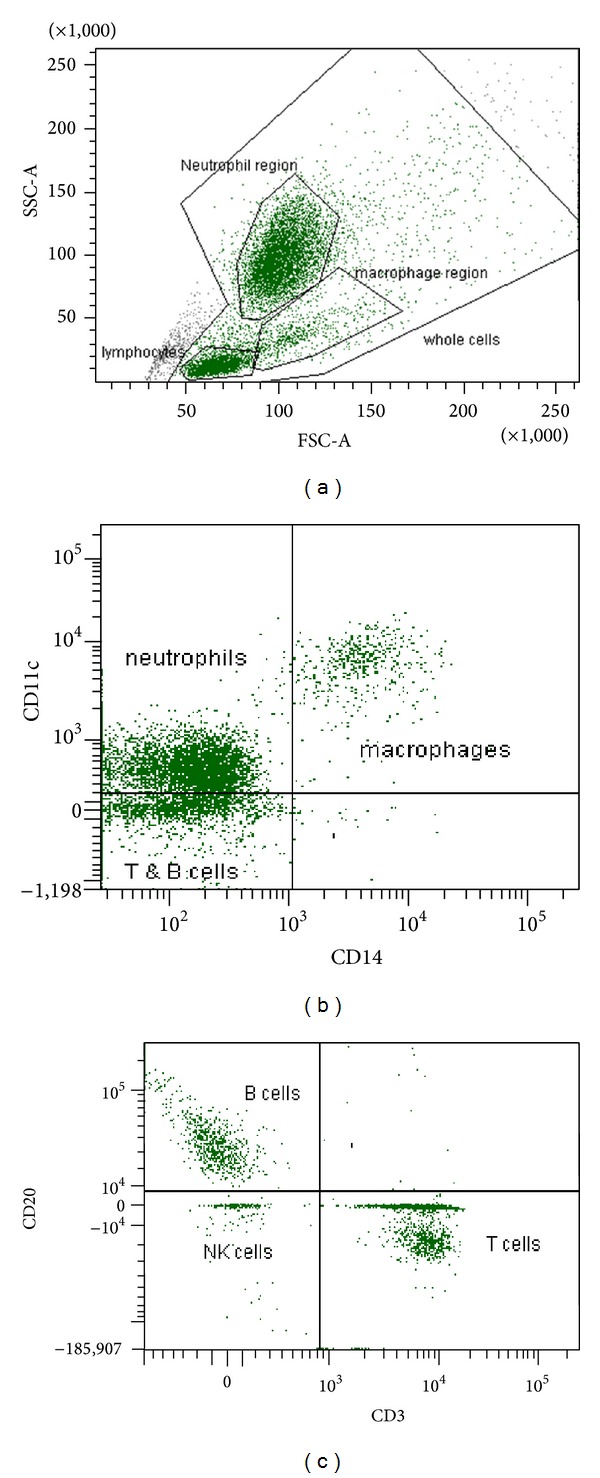
Flow cytometry plots. (a) Typical scatter profile from naïve marmoset blood showing the difference in size (FSC) and granularity (SSC) of the basic cell types. (b) Expression of CD11c and CD14 on monocytes/macrophages and neutrophils and (c) CD20 and CD3 expression on lymphocytes.

In order to fully characterise the immune response to infectious agent in the marmoset, single cell suspensions of lung and spleen tissue were also examined in conjunction with the traditionally used blood cells. These tissue homogenates are of particular interest in relation to target sites of infection: the lung as the site of initial infection following an inhalational challenge and the spleen as a representative organ following a parental challenge. Cell types targeted during this analysis include cells important in the innate response (e.g., neutrophils, macrophages, and NK cells) and the adaptive response (T and B cells) with a view to determine the response to infection and vaccination and to derive immune correlates of infection/protection. Dapi was included as a nuclear marker to ensure that the initial gating included only intact cells. Basic cell types in blood were easily identified by measuring size (forward) and granularity (side) scatter (Figure 1(a)). Identification of cell types in tissue samples was more difficult as the scatter profiles are less clearly compartmentalized. The common leukocyte antigen (CD45) normally used to locate all leukocytes in human samples also worked well in marmoset blood but failed to provide relevant information in the tissue samples. Confirmation of neutrophil identification was done by nuclear morphology and macrophages were identified by their adherent nature in initial experiments (data not shown). Neutrophils were stained as CD11c dim CD14− and macrophages as CD11c + CD14+ regardless of tissue origin (Figure 1(b)).  Figure 1(c) shows the basic division of lymphocytes between T, B, and NK cells from a healthy blood sample.

**Figure 2:**
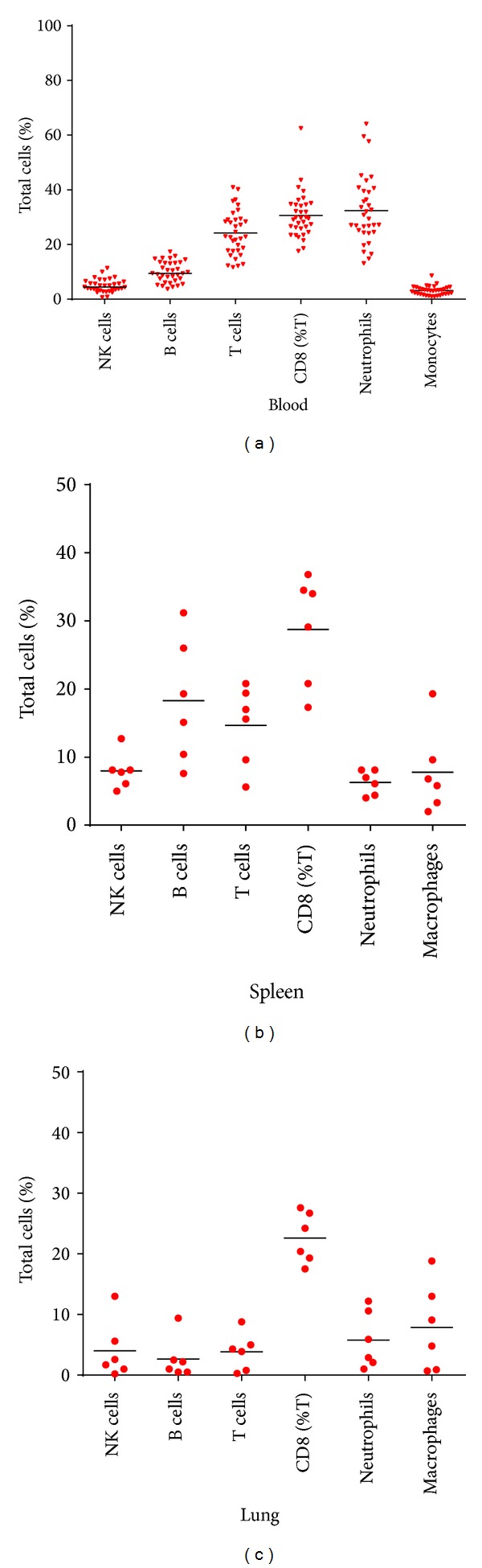
The percentage of the total leucocyte count for various cell types identified in naive marmosets (a) cells in naïve blood, (b) in naive spleens, and (c) in naive lungs. CD8 T cells are expressed as a percentage of CD3+ cells. Bar represents the median value.

Using this approach, the percentage of NK cells, B-cells, total T-cells, CD8+ T-cells, neutrophils, and monocytes was determined in the blood of naive marmosets (Figure 2(a), Table 1); approximately 63% of all lymphocytes were T cells, 25% B cells, and 12% NK cells. The variability of the data is depicted in Figure 2(a) with the greatest variability observed in the proportion of neutrophils. There were no obvious differences attributable to age or sex of the animals. This analysis was also applied to lung and spleen homogenates from naive marmosets (Figures 2(b) and 2(c)). Greater variability was observed in the data relating to the identification of cell types in tissue samples, attributed to the inherent difficulties in identifying cell types in tissue homogenates by size and granularity and also the smaller cohort of animals. As expected, low numbers of neutrophils are found in naive spleen or lung tissue (8% both). Healthy mouse spleens typically have approximately 1-2% granulocytes [30]. Understandably, there are few reports on the typical cell percentages expected in healthy human individuals for these tissues. However, it is reported that B cells are more prevalent in the spleens of humans at a ratio of 5 to 4 B to T cells than in the lungs which have a ratio of 1 to 8 B to T cells [34]. In marmoset data reported here, a ratio of 2 to 3 B to T cells in the spleen and 1 to 6 B to T-cells in the lungs was observed compared to a ratio of 3 to 2 B to T cells in mouse spleens [30].

Upon comparison, the marmoset data is generally consistent with previously reported data which is only available for marmoset blood samples [27] and information available for human blood [32, 33] (Table 1). However, one report found the proportion of CD8+ T-cells was almost three times greater in marmosets than humans, 61% to 21% respectively [35] compared to the 30% observed in this study and the work previously reported by Brok et al. [27]. Brok's study involved a small number of animals (eight) and also used a different CD8+ clone to identify cells. Contrastingly, in mice, differences are observed in the proportion of both B cells and neutrophils [31], although these differences are highly strain specific. C57BL/6J mice are reported to have 67% B cells and BALB/C mice 46%; both of which are consistently higher than the percentage found in marmosets and humans of approximately 25% (Table 1) [27, 31]. The proportion of neutrophils found in the blood of C57BL/6J mice at 13% is lower than the 35% found in marmosets and the 40–75% expected for healthy human blood. This is encouraging as neutrophils play a pivotal role in the innate response to infection [36]. A cross-species comparison suggests that monocytes comprise 3% of leukocytes (Table 1).

Levels of circulating cytokines and chemokines (IL-6, IL-1*β*, MIP-1*β*, MCP-1, Rantes, TNF*α*, and IFN*γ*) were also quantified in the blood, lung, and spleen of naïve marmosets from the Dstl colony. None of these cytokines were detected in blood samples from uninfected animals; however low levels of MIP-1*β*, MCP-1, and Rantes were found in spleen and lung tissue.

Preliminary investigation of the immune response has supported the development of marmoset model of infection at Dstl. The levels of different cell types were measured at specific times after challenge with inhalational* F. tularensis, B. pseudomallei,* and Marburg virus [13–15]. Following challenge with* F. tularensis,* increasing levels of NK cells, neutrophils, T cells, and macrophages were observed, peaking at 48 hours after challenge before rapidly declining. This study also demonstrated the importance of investigating the immunological response in key target organs, as an increase in CD8+ T cells and *γδ* T cells was observed in the spleen and lungs but not in the blood. Increasing levels of various cytokines, MCP-1, MIP-1*α*, MIP-1*β*, IL-6, and IL-1*β*, were observed in the lungs, spleen, and blood as the disease progressed (TNF*α* and IFN*γ* were not measured in this study).

Following inhalational challenge of marmosets with* B. pseudomallei*, an increase in the number of neutrophils was observed in the blood at 36 hours after challenge, followed by a rapid decline that was associated with an influx of neutrophils into the lung at 46 hours after challenge. A subsequent decline in the number of neutrophils in the lung was associated with the increased number in the spleen of animals that exhibited severe disease and were humanely killed. There was a gradual increase in the number of macrophages in the spleen as the disease progressed with numbers of macrophages peaking in the blood and lungs at 36 hours after challenge. A rapid decline in the number of macrophages in the lungs and blood was observed by 46 hours after challenge.

The levels of various cell types and cytokines were also measured in the blood of animals following inhalational challenge with Marburg virus [15]. In these animals a general increase in the numbers of T cells, NK cells, macrophages IFN-*γ*, IL-1*β*, and MCP-1 was observed with time (TNF*α* was not measured).

In order to gain more information from these acute bacterial infection models, we have sought out other markers from the literature. Primarily this was from marmoset models of autoimmune disorders such as rheumatoid arthritis and multiple sclerosis where the cross-reactivity of human antibodies was investigated, as well as the functionality of cells [37–40]. More recent work at Dstl has reported further cross-reactivity between marmoset cells and human cytokines to induce activity in marmoset T cells [36, 41]. These studies, combined with increasing information available on the cross-reactivity of human antibodies to various NHPs (e.g., NIH NHP reagent resource, http://www.nhpreagents.org/NHP/default.aspx), has expanded the ability to assess activation markers for disease. Detection of the following cell surface markers with human antibodies was trialed: CD54 (ICAM-1) associated with cellular adhesion, inflammation, and leukocyte extravasation; CD69 the early activation marker; CD16 as a macrophage activation marker; CD163 the alternative macrophage activation marker; and MHC class II (HLA-DR). CD56 was originally included to identify NK cells; however, it was noted that its expression on T cells was upregulated during disease and that cells defined as CD3+ CD16− CD56+ have been shown to be functionally cytotoxic in marmosets [37, 42].

**Figure 3:**
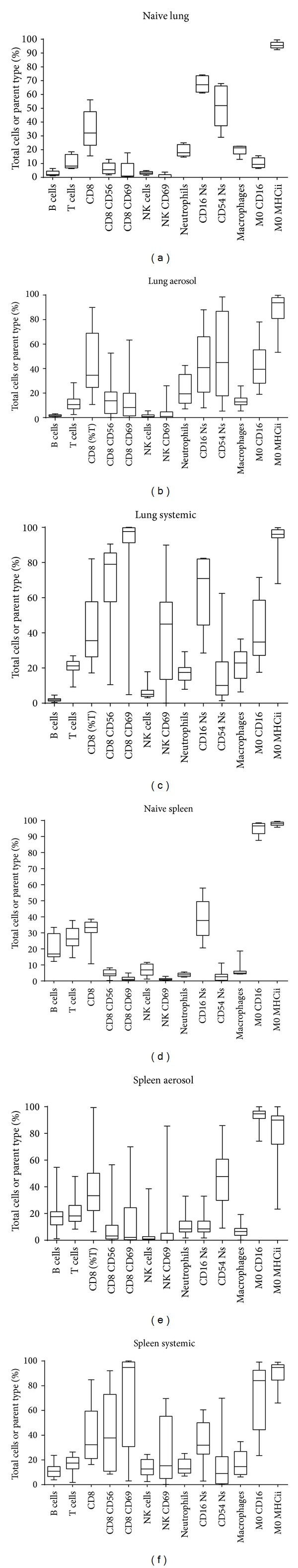
Cell types and activation markers from naïve and after an acute bacterial infection in spleen and lung tissues. Samples were taken at the humane endpoint approx. 4 days after challenge. B, T, NK cells, neutrophils, and macrophages expressed as percentage total whole cells, activation markers as percentages of parent cell type. (a) Naïve lung, (b) lung after aerosol challenge, (c) lung after systemic challenge, (d) naïve spleen, (e) spleen after aerosol challenge, and (f) spleen after systemic challenge.

These markers have been used to expand on our previously published work to determine changes in the activation status of basic cell types in response to an acute bacterial infection. Animals were challenged with bacteria at a comparable dose either by inhalation (*n* = 22) or by a systemic route (*n* = 12) and humanely killed once they had reached a humane endpoint (between day 4 and day 5 after challenge).  Figure 3 illustrates the cellular activity in representative tissues following inhalational (Figures 3(b) and 3(e)) or systemic challenge (Figures 3(c) and 3(f)) and in naïve samples (Figures 3(a) and 3(d)). Naïve T and NK cells appear to have similar resting activation states regardless of origin, whereas neutrophils and macrophages have differential expression of activation, for example, CD16. In response to disease, the proportions of the cell types appear to remain relativity constant; however, the activation markers provide more detailed information and show involvement of all the cell types explored. Extensive activation was to be expected considering that the samples were taken at the humane endpoint. There is also extensive variation between the samples from the infected animals, again indicative of the late time point in infection.

The spleen was chosen as a representative organ of systemic disease, and the cell activity shows that it is more actively involved in the systemic form of the disease with extensive activation in T cells, NK cells, and neutrophils. In the pneumonic form of disease, only the neutrophils and macrophages show changes in median values.

The response to infection within the lungs has similarities across disease routes in terms of neutrophil reduced expression of CD16 and CD54 and macrophage increased expression of CD16 and reduction in MHCII. Unexpectedly, the T and NK cells appear to be more actively involved in systemic disease, indicating that the disease develops a pneumonic element regardless of initial route of infection.

Levels of circulating cytokines and chemokines (IL-6, IL-1*β*, MIP-1*β*, MCP-1, Rantes, TNF*α*, and IFN*γ*) were also quantified in the lung and spleen samples. All of the cytokines (with the exception of Rantes) were expressed at high levels (ng/mg) in all samples, which was expected as the animals had succumbed to terminal disease.

## 4. Conclusion

The work presented here adds significant relevant information to the marmoset models of infection and to the understanding of the immune response in these animals. This work extends marmoset immunology from autoimmune disorders into the field of infectious diseases; this coupled with an increase in the information available on cross-reactivity of human reagents to a variety of NHPs increases the utility/application of marmosets as models of human disease. In conclusion, the immune response in marmosets to infectious disease can be characterised in terms of the phenotype and activation status of all the major immune cells and key cytokine and chemokine expression. This can aid in the identification of correlates of infection or protection in medical countermeasures assessment studies. This information can also potentially be used for pivotal studies to support licensure of products under the FDA Animal Rule.

This, in conjunction with the small size of marmosets, their immune response to infection that is comparable to humans, and the ability to house more statistically relevant numbers within high containment, makes the marmoset an appropriate animal model for biodefense-related pathogens.

## Figures and Tables

**Table 1:** Comparison of the percentages of different cell types observed in the blood from healthy marmosets, mice, and humans.

Cell type	Identification markers	Marmoset (present data)	Reported percentage observed in blood (%)			
			Marmoset [27]	Mouse^4^ [30, 31]	Human Asian [32]	Human Caucasian [33]
Number of samples		130+	20		230	200+
^ 1^B cells	CD20+ CD3−	25 (10–45)	10–25	60 (21–85)	18	7–23
^ 1^NK	CD20− CD3− CD56+	12.5 (2–30)	25–50	nd	15	6–29
^ 1^T cells	CD20− CD3+	62.5 (25–90)	50–75	49 (24–99)	67	61–85
^ 2^CD8+ T cells	CD20− CD3+ CD8+	30 (20–65)	25–50	30 (24–37)	27	15–40
^ 3^Neutrophils	CD11c dim CD14−	35 (20–65)	nd	13 (8–16)	nd	nd
^ 3^Monocytes	CD11c dim CD14−	3 (1–10)	nd	1-2	nd	nd

^1^Reported as percentage of lymphocytes.

^2^Reported as percentage of T cells.

^3^Reported as percentage of leukocytes.

^4^Recalculated as average mouse values from reported strains.

nd: not determined.
